# Ear necrosis syndrome in weaning pigs associated with PCV2 infection: A case report

**Published:** 2012

**Authors:** Vassilis Papatsiros

**Affiliations:** Department of Medicine (Clinic of Farm Animal Medicine), Faculty of Veterinary Medicine, University of Thessaly, Karditsa, Greece.

**Keywords:** Circovirus, PMWS, Ear necrosis, Pig, Vaccine

## Abstract

Porcine necrotic ear syndrome (PNES) in pigs has been reported as an increasing health problem in many countries with intensive pig farming. The etiology of this disease is complex and the presumed triggering factors can be divided into infectious and non-infectious agents. The present report describes a case of Porcine Circovirus type 2 (PCV2), infection associated with lesions of PNES at the weaning stage of a farrow-to-finish pig farm. Approximately 35% of weaners (1-3 weeks after weaning) presented clinical symptoms similar to Post-weaning Multisystemic Wasting Syndrome (PMWS). About 2-3 weeks after weaning the first lesions of PNES occurred in approximately 20% of pigs, resulting in a significant health problem characterized by poor growth or severe wasting and finally mortality up to 15% in some batches. Moreover, approximately 5% of survived weaners, during growing / finishing stage, presented poor growth and secondary co-infections that lead to death. The present study based on the clinical signs, serological and pathological examinations, indicates that weaners suffered by sub-acute PCV2 infection resulting in PMWS associated with PNES. The lesions of PNES were initially observed at the same period (4-8 weeks of age) with the higher seroprevalence of PCV2 infection. Metaphylaxis of this case included intramuscular injection of florfenicol for the treatment and control of skin lesions and respiratory signs. Moreover, piglets were vaccinated against PCV2. In conclusion, sub-acute PCV2 infection could be included in triggering factors PNES in weaners. The mass vaccination against PCV2 of infected piglets might be effective in reduction of clinical signs and losses of PNES in cases of PCV2 infection associated with PNES.

## Introduction

Necrotic ear syndrome or ear necrosis in pigs has been reported as an increasing health problem in many countries with intensive pig farming.^1^ It is characterized by large erosive lesions at the margin of the pinna(e) in both sexes. It occurs mainly in weaning pigs and growers/ fatteners with bodyweight ranging approximately from 10 to 40 kg.^2^ The earliest lesions are normally visible on the ear’ tips at 6-7 weeks old pigs, beginning as a superficial vesicular dermatitis associated with superficial auricular trauma, which can bleed, attractive to pen mates, who may then start to bite at the lesion, resulting in swelling and reddening of the ear. Localized lesions slowly healed or sporadically progressed to deep necrotic ulcers, cellulitis, vasculitis, thrombosis, ischemia.^3^

The etiology of this disease is complex and therefore it is often named as porcine ear necrosis syndrome (PENS). The presumed triggering factors can be divided into infectious and not infectious agents. It is usually the result of a mixed infections causing damage to the skin. *Staphylococcus hyicus *is the most common isolated agent in lesions of PENS cases, but other pathogens such as *Mycoplasma suis*, *Streptococcus suis* and spirochetes are often implicated.^4^^-^^8^ Moreover, non-infectious factors such as intensive pen density and overpopulation, poor air quality with high concentrations of gases (e.g. ammonia), poor hygienic conditions, copper and magnesium deficiency, contamination of feed with mycotoxins and cannibalism, were associated with an increased risk of incidence of PENS.^2^^,^^9^^-^^12^

Recently, an important causative role has also been attributed to immunosuppressive agents such as Porcine Circovirus type 2 (PCV2), Porcine Reproductive and Respiratory Syndrome Virus (PRRSV), as well as mycotoxins.^7^^,^^13^ PCV2 is considered to be involved in etiology agents of the development of PNES^14^ and is present in ear lesions PCV2 infected pigs,^15^ associated sometimes with co-infections of PRRSV,^16^^,^^17^ *Pasteurella multocida*, *Streptococcus suis* types 1 and 2 and other pathogens.^18^^,^^19^

Last years a marked increase of field cases characterized by PNES associated with PCV2 infection has been observed in the USA,^20^ in Canada^21^ and Europe.^1^^,^^9^^,^^14^^,^^22^ In Greece, during the last two years our clinical observations in field conditions suggest the cases of PNES have been increased and when Porcine Circovirus Associated Diseases (PCVAD) is present on a farm, more pigs with ear tip necrosis are observed, accompanied with severe wasting, respiratory clinical signs and significant mortality.^23^

## Case Description

**History** ***.*** The present report describes a case of PCV2 infection associated with lesions of PNES at the weaning stage of a farrow-to-finish pig farm of 200 sows (Large x White x Landrace) in Central Greece. Weaning took place at the age of 25 ± 3 days. All weaning pigs were moved every week into the flat deck unit, grouped in pens of 15 pigs. This farm applied all appropriate facilities of biosecurity of good hygiene.

**Clinical observations. **Approximately 35% of weaning pigs (1-3 weeks after weaning), through out of different batches, presented clinical symptoms similar to Post-weaning Multisystemic Wasting Syndrome (PMWS), such as fever, anorexia, diarrhea, considerable weight loss cough, dyspnea, paleness of the skin, enlarged lymph nodes and lethargy. About 2-3 weeks after weaning the first lesions of PNES occurred in approximately 20% of pigs, resulting in a significant health problem characterized by poor growth or severe wasting and finally mortality up to 15% in some batches. Moreover, approximately 5% of survived weaning pigs, during growing / finishing stage, presented poor growth and secondary co-infections that lead to death. 

**Fig. 1:**
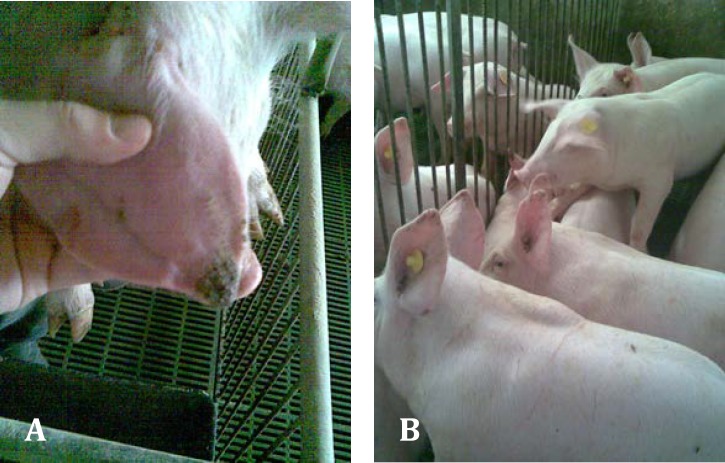
**A.** Weaning pig with initial lesions of PNES. **B.** Group of pigs with PNES clinical signs

**Fig. 2 A and B:**
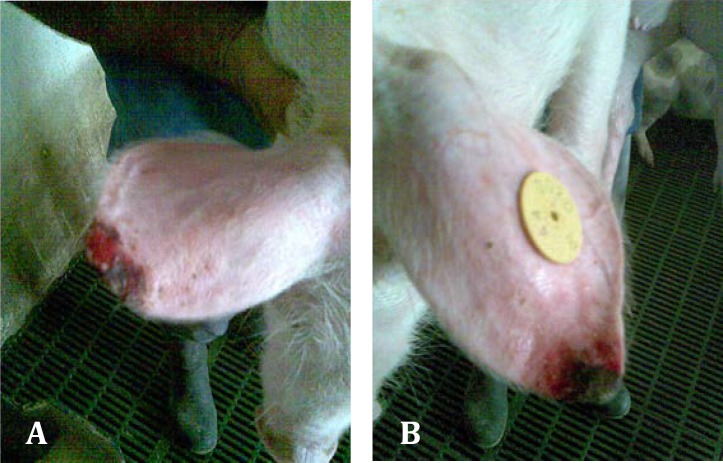
Weaning pigs with severe lesions of ear necrosis syndrome

Regarding the lesions of PNES, they initially appeared at the margin of the pinna(e) (on the tip of the ear or at its base) as a superficial vesicular dermatitis associated with superficial auricular trauma (Fig. 1. A and B). These lesions were characterized by necrosis, vasculitis, dry gangrene and inflammation that sporadically progress in exudative or ulcers (Fig. 2. A and B). In addition, the necrotic lesions were complicated with secondary infections.

**Sampling / Diagnosis. **Blood samples were collected from pigs at age of 5-6 weeks (group 1), 7-8 weeks (group 2), 9-10 weeks (group 3) and 22-23 weeks (group 4). Serum samples were stored at -20 ^°^C, and analyzed for PCV2 IgM and IgG antibodies by Ig capture ELISA based on method described by van Esch and Wellenberg.^24^ IgM and IgG specific ELISA tests (Ingezim PCV IgG / IgM^®^, Spain) were also used. Moreover, five weaning pigs (4-9 weeks of age) were euthanized for necropsy. Samples collected from lung, heart, inguinal lymph node, tonsil, thymus, spleen, small and large intestines, liver, kidney, and pancreas were fixed in 10% (w/v) buffered formaldehyde for 24 h and embedded in paraffin by standard histologic procedures.

## Results

The serological results for PCV2 IgM and IgG responses in sera blood samples are shown in Table 1. Based on the serological results and the proposed interpretation of PCV2 IgM and IgG ELISA results in pigs,^24^ it is noticed that infected pigs suffered by sub-acute infection.

**Table 1:** Percentage of PCV2- positive pigs (PCV2 IgM, IgG) in different ages

**Animals**	**Test system**	**No.**	**Percentage positive pigs**
Group 1 (5-6 weeks)	IgM ELISA IgG ELISA	10 10	60% (6/10) 20% (2/10)
Group 2 (7-8 weeks)	IgM ELISA IgG ELISA	10 10	50% (4/10) 10% (1/10)
Group 3 (9-10 weeks)	IgM ELISA IgG ELISA	10 10	45% (4/9*) 10% (1/10)
Group 4 (22-23 weeks)	IgM ELISA IgG ELISA	15 15	21% (3/14*) 7% (1/15)

* insufficient serum. No. = number of animals.

Pathological examinations based on microscopic analysis of lymphoid tissue immunohistochemistry and in situ hybridization, indicated abundant amount of PCV2 DNA within observed histopathological PMWS-specific lesions (lymphocyte depletion in lymphoid tissues and interstitial pneumonia). 

**Treatment protocol / Control strategies. **In this case of PCV2-infection associated with PNWS, intramuscular injection of florfenicol (20 mg kg^-1^ body weight) was suggested for the treatment and control of skin lesions and respiratory signs. Moreover, the following batches of piglets were vaccinated against PCV2 with commercial vaccine Porcilis^®^ PCV (MSD Animal Health Animal Health) administered with 2 × 2 mL dose scheme (first dose at 7^th^ day of age, with the second dose 3 weeks later -day of weaning). This vaccination scheme resulted in a significant reduction of PNES prevalence and mortality rate of weaning pigs in the herd (data not shown). At the same time, for these following batches of piglets a grouping of 10 animals per pen at weaning stage was applied.

## Discussion

Based on the clinical signs of infected weaning pigs and serological as well as pathological results, we concluded that weaning pigs suffered by sub-acute PCV2 infection resulting in PMWS associated with PNES. The lesions of PNES were initially observed at the same period (4-8 weeks of age) the higher seroprevalence of PCV2 infection, as is shown in Table 1. 

The results of the present study indicate that PNES co-existing with PCV2 infection in many cases. The prevention and control of PCVAD are based on proper immunization (vaccination) and management practices.^19^ Nowadays, at least four commercial vaccines are available against PCVAD in piglets and sows. The vaccines have succeeded in reducing losses caused by PCV2 in Europe, Canada and the USA.^25^ The findings of our study agree with the results of previous studies,^1^^,^^14^^,^^22^ with the difference that we managed to reduce the losses due to PCV2 infection associated with PNES, using an inactivated vaccine in 2 doses scheme (early at 7^th^ day of age and at day of weaning).

In conclusion, sub-acute PCV2 infection could be included in triggering factors PNES in weaners. The mass vaccination against PCV2 of infected piglets might be effective in reduction of clinical signs and losses of PNES in cases of PCV2 infection associated with PNES.
